# PP4397/FlgZ provides the link between PP2258 c-di-GMP signalling and altered motility in *Pseudomonas putida*

**DOI:** 10.1038/s41598-018-29785-w

**Published:** 2018-08-15

**Authors:** Lisa Wirebrand, Sofia Österberg, Aroa López-Sánchez, Fernando Govantes, Victoria Shingler

**Affiliations:** 1 Department of Molecular Biology, Umeå University, SE-901 87, Umeå, Sweden; 2 Departamento de Biología Molecular e Ingeniería Bioquímica, Universidad Pablo de Olavide, Sevilla, Spain

## Abstract

Bacteria swim and swarm using rotating flagella that are driven by a membrane-spanning motor complex. Performance of the flagella motility apparatus is modulated by the chemosensory signal transduction system to allow navigation through physico-chemical gradients – a process that can be fine-tuned by the bacterial second messenger c-di-GMP. We have previously analysed the *Pseudomonas putida* signalling protein PP2258 that has the capacity to both synthesize and degrade c-di-GMP. A PP2258 null mutant displays reduced motility, implicating the c-di-GMP signal originating from this protein in control of *P. putida* motility. In *Escherichia coli* and *Salmonella*, the PilZ-domain protein YcgR mediates c-di-GMP responsive control of motility through interaction with the flagellar motors. Here we provide genetic evidence that the *P. putida* protein PP4397 (also known as FlgZ), despite low sequence homology and a different genomic context to YcgR, functions as a c-di-GMP responsive link between the signal arising from PP2258 and alterations in swimming and swarming motility in *P. putida*.

## Introduction

Like many other bacteria, Pseudomonads swim and swarm using rotating flagella powered by membrane ion gradients to relocate to environments optimal for their metabolism^1^. Bacteria can control flagellar-driven motility in a number of ways, including through control of flagella assembly^2,3^ and taxis signal-transduction pathways that alter the direction of flagella rotation^4^. One of the most recently identified means of control is through the second messenger cyclic di-GMP [bis-(3′–5′)-cyclic dimeric guanosine monophosphate; c-di-GMP]. In addition to motility control, this near ubiquitous bacterial second messenger is also involved in co-ordinating developmental processes, regulation of virulence determinants, and the transition to biofilm formation^5–7^.

In Pseudomonads, low intracellular levels of c-di-GMP are associated with a motile (flagellated) planktonic mode of growth, while elevated levels sequentially trigger slowing down of flagella motility for surface attachment, and production of adhesins and biofilm components for a consequent sessile lifestyle^8–10^. Diguanylate cyclases (DGCs) and phosphodiesterases (PDEs) – which control the dynamic changes in the intracellular levels of this second messenger, are abundant in most bacteria. DGCs contain a GGDEF motif within their catalytic A-site and synthesise c-di-GMP from two molecules of GTP; conversely, PDEs degrade c-di-GMP – either to linear pGpG [EAL-motif proteins] or to two molecules of GMP [HD-GYP motif proteins]. Although DGCs and PDEs mediate opposing functions, they are often linked together in multi-domain proteins. In most cases, however, one of the domains has lost its catalytic capacity and instead has been adapted to regulate the function of the protein^11^. So far only a few proteins have been shown to be *bona fide* dual functional proteins that possess both c-di-GMP synthesising and degradative activities. One such protein is the motility associated c-di-GMP signalling protein PP2258 of *Pseudomonas putida*^12^. Lack of PP2258 (or its over-expression) results in decreased motility due to elevated c-di-GMP levels, implying that c-di-GMP dependent signalling from PP2258 is involved in motility control of *P. putida*^12,13^.

The enzymatic processes that make and break c-di-GMP are fairly well understood. However, the upstream signals that control the activities of DGCs and PDEs, and the downstream target effector proteins (and RNAs) that respond to c-di-GMP signalling have not been as extensively elucidated^14^. One common domain of c-di-GMP responsive effector proteins is the PilZ domain. This type of domain undergoes conformational changes upon c-di-GMP binding, which leads to alterations in protein-protein interactions and allosteric affects. However, differences exist between individual PilZ domains when it comes to binding mode, stoichiometry, and quaternary structure^15,16^. In the enterics *Escherichia coli* and *Salmonella*, the PilZ domain protein YcgR co-localizes with flagella and acts as a brake in response to c-di-GMP by interacting with components of the flagella motor^17–19^. In these organisms, the non-rotating part of the motor – the stator – is formed by membrane complexes of the MotA/MotB proteins that provide the ion-translocating channels that energise the rotor. The stator lies above the FliG/FliM/FliN rotary switch complex, which drives rotation of the flagellum and also sets the rotational direction. The carboxy-PilZ domain of YcgR binds c-di-GMP with a 1:1 ratio and binding of c-di-GMP causes YcgR to adopt a more condensed conformation without altering its monomeric status^20^, while it’s amino-terminal domain is involved with interactions with its targets – MotA, FliG and/or FliM – to result in reduced torque and motility^17–19^.

*Pseudomonas aeruginosa* and other Pseudomonads have an additional set of Mot proteins – MotC/MotD^21,22^ – which in the case of *P. aeruginosa* appear to be the predominant complexes involved in swarming and swimming motility^23^. Pseudomonads also encode a distant relative of YcgR – FlgZ – that affects swimming motility of *P. fluorescens* and *P. putida*^24^. Most recently, the *P. aeruginosa* PA14 FlgZ counterpart has been shown to interact with MotC (rather than MotA) to control its swarming motility^23^. The structure and c-di-GMP binding properties of the *P. putida* KT2440 FlgZ counterpart – PP4397 – have previously been determined; unlike YcgR, PP4397 undergoes a dimer-to-monomer transition upon c-di-GMP binding *in vitro*^25^. Hence, in addition to low sequence identity, FlgZ/PP4397 exhibit disparate biochemical properties to those of YcgR.

Despite detailed structural studies, little is known about how expression of *P. putida* PP4397 is controlled, what upstream protein(s) can control its c-di-GMP responsive activity, and if it is a genuine functional counterpart of YcgR. In this work we provide evidence that expression of PP4397 is controlled by two alternative σ-factors – σ^54^ and the flagella specific σ^FliA^ – to allow coupling of PP4397 expression to other flagella motility related genes. Furthermore, we show that despite very limited amino acid sequence identity and different *in vitro* properties to those of YcgR, FlgZ/PP4397 lies downstream of PP2258 in c-di-GMP responsive motility control in *P. putida* and can be functionally replaced by YcgR in this process.

## Results and Discussion

### pp4397/flgZ is co-transcribed with flagellar associated genes dependent on σ^54^ and σ^FliA^

**Figure 1:**
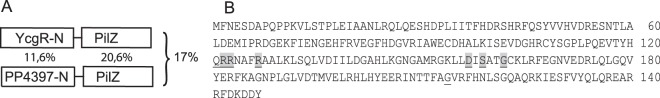
PP4397 exhibits limited sequence identity with YcgR but maintains key residues involved in c-di-GMP binding. (**A**) Schematic illustration of the domains and % identity of YcgR and PP4397/FlgZ as detailed in the text. (**B**) The amino acid sequence of PP4397 is shown with nine highly conserved residues of PilZ domains (highlighted in bold and underlined) that encompass the RXXXR and (D/N)XSXXG motifs of c-di-GMP binding-proficient type I PilZ domains^34^. Residues, which when substituted by alanine, essentially abolish c-di-GMP binding by PP4397/FlgZ^25^ are highlighted in grey. A complete alignment of PP4397 and YcgR as in^15,25^ using ESPript^35^ is shown in Fig. S1, while the extensive homology with FlgZ proteins of representative Pseudomonads is shown in Fig. S2.

**Figure 2:**
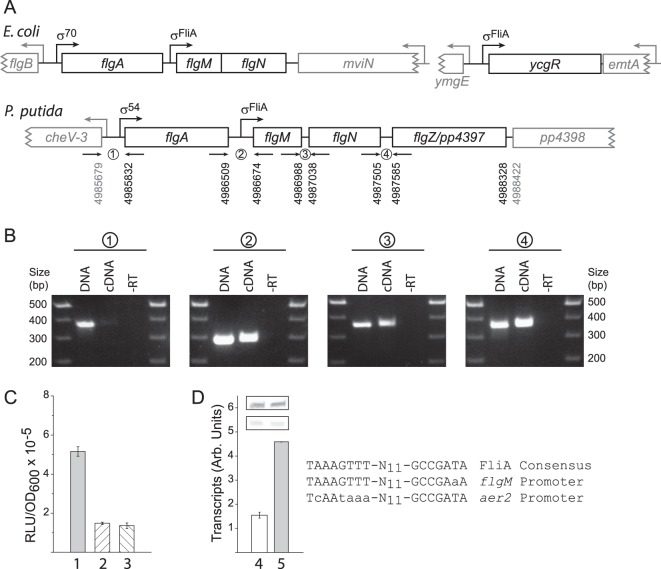
Transcription of PP4397 is dependent on both σ^54^ and σ^FliA^. (**A**) Comparison of the genomic context of *E. coli ycgR* and *P. putida flgZ*/*pp4397* genes. Upper schematic, illustration of *E. coli* MG1655 *flgA, flgMN* and *ycgR* genes (shown in black) with their cognate promoters indicated. Divergently transcribed genes are shown in grey. Lower, similar schematic of the *P. putida flgA, flgM, flgN-*like *pp4396* and *flgZ*/*pp4397* genes shown in black and the divergently transcribed *cheV-3* and *pp4398* genes shown in grey. The locations of primer pairs used for analysis of co-transcription of genes as depicted in panel B are indicated. (**B**) Agarose gels of PCR reactions using the indicated primer pairs (panel A) on *P. putida* genomic DNA, cDNA, or control samples where no reverse transcriptase was added to the cDNA reaction mixture (-RT). (**C**) *In vivo* transcription from P_*flgM*_ in wild type *P. putida* KT2440 (1) and its FliA null (2) and RpoN null (3) counterparts carrying mono-copy transcriptional fusions to the promoter-less *luxAB* reporter genes (PP3733 to PP3735, Table S1). Graphed values are the average +/− standard deviation of six independent determinations from cultures grown to the stationary phase in LB (OD_600_ ~5.0 for wild-type and FliA null; ~2.1 for RpoN null). (**D**) Single-round *in vitro* transcription assays using 10 nM supercoiled DNA templates harbouring the σ^*FliA*^ –dependent *P. putida* P_*aer2*_ promoter (4; pVI1011) or P_*flgM*_ (5; pVI2368) in the presence of 10 nM σ^FliA^-RNAP. Inset shows images from one of two independent experiments used to obtain the graphed average values (P_*flgM*_ upper; P_*aer2*_ lower). A comparison of the P_*aer2*_ and P_*flgM*_ promoter sequences with the optimal consensus^27^ for *P. putida* σ^FliA^ is shown to the right.

*In silico* searches of genome sequenced *P. putida* KT2440 identified PP4397/FlgZ as the protein exhibiting highest identity to YcgR (17% and 24% identity with *E. coli* and *Salmonella*, respectively). Despite this low homology, similar to YcgR, PP4397/FlgZ also possesses a type I c-di-GMP binding PilZ and PilZN-like domain (Figs 1 and S1). The assembly and operation of Pseudomonad flagella depends on more than 50 genes arranged in clusters that are controlled by a four-tiered transcriptional regulatory cascade^26^. As depicted in Fig. 2A (upper), enteric *ycgR* lies in a monocistronic operon, while the *P. putida flgZ/pp4397* gene lies within a cluster of genes encoding proteins involved in flagellar biosynthesis (Fig. 2A, lower). We therefore wanted to clarify if *pp4397* is co-transcribed with any of these genes.

Reverse transcription polymerase chain reaction (PCR) assays with RNA isolated from *P. putida* KT2440 and primer pairs spanning *pp4397* and adjacent genes showed that *pp4397* is co-transcribed with the σ^FliA^ anti-σ-factor gene *flgM* and *pp4396* – a gene encoding an FlgN family protein (Fig. 2B). An appropriately located σ^FliA^-dependent promoter is found immediately upstream of the *flgM-flgN-flgZ/pp4397* genes cluster (see Fig. 2A). Hence, this data is consistent with the idea that *pp4397* lies within a tri-cistronic *flgM-flgN-pp4397* operon, but does not exclude the possibility that internal promoters within *flgM* and/or *flgN* may also contribute to transcription of *pp4397*. Recent analysis of transcription of *pp4397*/*flgZ* counterparts in *P. fluorescens* F113 and *P. putida* KT2440 could only document a partial dependence on σ^FliA^ as assessed using FliA null strains^10,24^. Therefore, we extended the analysis to include the upstream *flgA* gene that encodes a protein involved in flagellar P-ring formation. As shown in Fig. 2B, transcription of the *flgM-flgN-pp4397* operon also appears to be mediated by read-through transcription from a σ^54^-dependent promoter located upstream of the *flgA* gene.

To further substantiate the above findings, we performed *in vivo* and *in vitro* transcription assays. *In vivo* transcription of *pp4397*/*flgZ* was monitored using a *luxAB* (luciferase) transcriptional fusion generated downstream of *pp4397* in wild-type, FliA null, and RpoN (σ^54^) null *P. putida* backgrounds. Consistent with co-dependence on both these σ-factors, transcriptional output was decreased but not abolished in both of the null strains as compared to wild-type when grown Luria-Bertani (LB) broth (Fig. 2C). Functionality of the identified σ^FliA^ promoter located upstream of the *flgM-flgN-pp4397* tri-gene cluster (P_*flgM*_) was verified by single-round *in vitro* transcription assays with σ^FliA^-RNA polymerase reconstituted from purified *P. putida* components (Fig. 2D). As anticipated by its high identity to the optimal consensus for *P. putida* FliA dependent transcription, the near consensus *flgM* promoter produces high levels of transcripts as compared to the previously analysed suboptimal σ^FliA^-dependent promoter for *aer2*^27^.

The difference in σ-dependence for the *flgA* promoters in Pseudomonads as compared to enterics is due to differences in the hierarchical expression of flagellar genes in these two species. Transcription of genes needed early, e.g. *flgA*, are dependent on σ^54^ in *Pseudomonas* and σ^70^ in *E. coli* and *Salmonella*^26,28^. Thus, while the genomic context of *pp4397/flgZ* is different from the moncistronic context of *E. coli ycgR* gene, transcriptional control of the *flgA* and *flgMN* counterparts is conceptually similar, with a promoter upstream of *flgA* generating read-through transcription of downstream genes within a σ^FliA^-dependent operon. Given that the *flgZ* gene is highly conserved in sequence and synteny in all sequenced Pseudomonads^24^, including *P. fluorescens* F113, *P. aeruginosa* PA14, and *P. aeruginosa* PAO1 (Fig. S2), co-dependence on both σ^FliA^ and σ^54^ as found here for *P. putida* KT2440 is likely the case for these and other Pseudomonads. It is interesting to note that this regulatory arrangement would ensure σ^54^-dependent transcription of the *flgM-flgN-pp4397* genes even in the absence of σ^FliA^. Therefore, it would also result in σ^FliA^-independent production of PP4397/FlgZ that serves to slow-down flagella (as detailed below) and production of the anti-σ^FliA^-factor FlgM to block new *de novo* flagella production – two steps needed for preparation to enter the biofilm mode of growth^9^.

### Lack of PP4397 results in altered swimming and swarming motility

**Figure 3:**
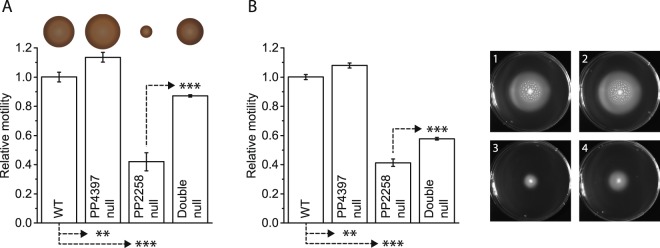
The reduced motility phenotypes of PP2258 null *P. putida* are partially rescued by the absence of PP4397. (**A**) Relative swimming motility of *P. putida* KT2701 wild type (WT) and the indicated null derivatives (Table S1) on 0.3% soft agar LB plates. Representative swim rings are shown above. (**B**) Relative swarming motility of the same strains on 0.5% agar plates. Representative swarm zones are shown to the right. Graphed values in all cases are averages with standard deviations calculated from three independent colonies and were normalized by setting the values of the wild type strain as 1. *P*-values shown for relevant comparisons were calculated with two-tailed student *t*-test (***P < 0.001; **P < 0.01).

Having established that PP4397 is co-ordinately regulated with genes of the flagella regulon, we next addressed its involvement in c-di-GMP responsive control of flagella-mediated motility. To test potential involvement of PP4397 in motility control, we first generated a null mutant of *P. putida* KT2701 (a streptomycin resistant derivative of the genome sequenced *P. putida* KT2440) in which the majority of the *pp4397/flgZ* gene was replaced by a tetracycline resistance cassette. When tested on rich (LB) 0.3% soft agar swimming motility plates or 0.5% agar swarming motility plates, the Δ*pp4397*::Tc strain displayed only a slightly enhanced motility phenotype as compared to the wild type (Fig. 3).

The above data is consistent with previous findings for *E. coli* and *Salmonella* in which a YcgR null counterparts only exhibited increased motility in a high c-di-GMP background – a condition that normally results in decreased motility^20^. Therefore, we also introduced the Δ*pp4397*::Tc allele in *P. putida* KT2701 Δ*pp2258*::Km, a previously analysed strain known to have elevated c-di-GMP levels and a swimming motility defect on soft agar plates despite having a wild type number of polar flagella^12^.

Both the Δ*pp2258*::Km and the Δ*pp2258*/Δ*pp4397* double mutant strains exhibit prolonged lag phases upon outgrowth from overnight cultures on rich media (LB), but once they attain exponential growth, have doubling times (41.3 +/− 2.9 min) similar to, but slower, than the wild type and the Δ*pp4397*::Tc strains (36.2 +/− 1.7 min; see Fig. S3A). While the exact level of c-di-GMP in the Δ*pp2258*::Km PP2258 null strain is unknown, elevated c-di-GMP levels in this strain and the Δ*pp2258*/Δ*pp4397* double null strain are insufficient to provoke altered biofilm production or dispersal phenotypes (Fig. S3B–D), as judged using a microtitre dish-based assays that employs serial dilution to recapitulate biofilm growth and dispersal kinetics^29^. As detailed in Fig. S3, in both cases biofilm production and dispersal rates appear similar to wild type, despite a delay as a consequence of growth kinetics.

In contrast, the reduced motility seen for the Δ*pp2258*::Km strain in both swimming and swarming abilities was significantly rescued in the double mutant [compare PP2258 null with the PP2258/PP4397 double null in Fig. 3A,B]. Even though exponentially growing cells were used for inoculation of the motility assay plates (see Methods) reduced growth rates as a consequence of elevated c-di-GMP levels probably, at least in part, underlies why full motility comparable to the wild-type strain could not be achieved. Taken together, the data in Figs 3 and S3 consolidate a role for PP4397/FlgZ of *P. putida* in swimming and swarming motility (but not biofilm production or dispersal), and provide the first evidence that PP4397/FlgZ functions downstream of PP2258 in response to modulation of c-di-GMP levels.

### The c-di-GMP binding property of PP4397 is required to mediate motility control

**Figure 4:**
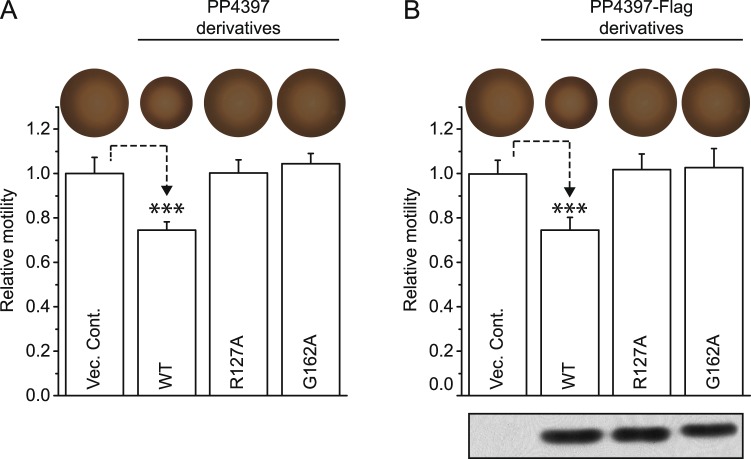
The function of PP4397 in motility control is dependent on its c-di-GMP binding ability. Relative swimming motility of the double PP2258/PP4397 null derivative of *P. putida* KT2701 on 0.3% soft agar LB plates supplemented with carbenicillin. (**A**) Strains harbouring either a vector control (Vec. Cont.; pVI2300) or *lacI*^Q^/P_*tac*_ expression plasmids for derivatives of PP4397: c-di-GMP binding proficient wild type PP4397 (WT, pVI2301), or c-di-GMP binding deficient mutants R127A (pVI2302) or G162A (pVI2303). (**B**) Strains harbouring either a vector control (Vec. Cont.; pVI2300) or *lacI*^Q^/P_*tac*_ expression plasmids for FLAG-tagged derivatives of PP4397: c-di-GMP binding proficient wild type PP4397 (WT, pVI2304), or c-di-GMP binding deficient mutants R127A (pVI2305) or G162A (pVI2306). Graphed values are averages with standard deviations calculated from three independent colonies. Experiments were normalized by setting the values of the double mutant harbouring the vector control as 1 and *P*-values calculated with two-tailed student *t*-test (***P < 0.001). Images of representative swim rings are shown above the graphed values. The insert in panel B shows Western analysis of the FLAG-tagged PP4397 derivatives present in 10 µg of crude extract from the same cells. The cropped Western analysis image is derived from the same gel and is shown alongside molecular size markers in Fig. S4A.

To verify that the phenotype for the Δ*pp2258*/Δ*pp4397* double null strain was not attributable to indirect effects on upstream genes within the *flgM-flgN-flgZ/pp4397* operon, this strain was complemented with plasmids carrying either a native version of the *pp4397/flgZ* gene or a C-terminally FLAG-tagged version under control of the IPTG inducible *lacI*^Q^/P_*tac*_ promoter. The *lacI*^Q^/P_*tac*_ system of the expression plasmid is leaky and produced sufficient PP4397/FlgZ to reverse the effect of lack of PP4397 in the double Δ*pp2258*/Δ*pp4397* strain – i.e. expression of PP4397 or PP4397-FLAG in the double mutants resulted in a reduced motility phenotype approximating that of the PP2258 null strain (Fig. 4). The motility phenotypes shown in Fig. 4 were unaffected by addition of 0.5 mM IPTG (data not shown). These results confirm that the motility rescue phenotype of the double mutant is due to lack of PP4397/FlgZ.

Biochemical analysis of PP4397 has shown that alanine substitutions of arginine 127 (R127A) or glycine 162 (G162A) both abolish the capacity of PP4397 to bind c-di-GMP. Arginine 127 is directly involved in binding of c-di-GMP, while glycine 162 is conserved among PilZ domain proteins and is probably needed for correct folding^25^. To confirm that c-di-GMP binding is required for PP4397 to exert its phenotypic effects, equivalent expression plasmids for native and C-terminally FLAG-tagged PP4397-R127A and PP4397-G162A derivatives were generated and tested as described for wild type PP4397. Neither of these c-di-GMP binding defective derivatives mediated a reduced motility phenotype (Fig. 4), even though they were expressed at the same levels as the wild type protein (Figs 4B and S4A). Thus, these results demonstrate that PP4397 is c-di-GMP responsive *in vivo*, and that c-di-GMP binding by PP4397/FlgZ is a prerequisite for its effects on *P. putida* motility.

### YcgR, like PP4397, restores a motility defect in *P. putida* Δpp2258/Δpp4397

**Figure 5:**
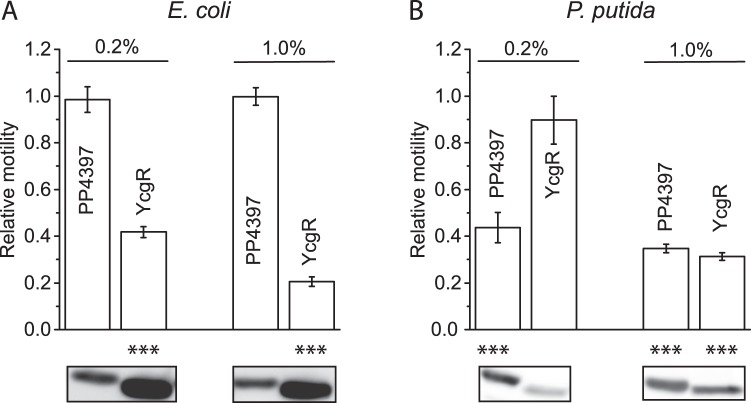
Cross-species complementation - both PP4397 and *E. coli* YcgR restore a motility defect to the double PP2258/PP4397 null derivative of *P. putida*. (**A**) Relative swimming motility of *E. coli* MG1655-Δ*yhjH/*Δ*ycgR* carrying *araC*/P_*BAD*_ expression plasmid for *P. putida* PP4397-FLAG (pVI2370) or *E. coli* YcgR-FLAG (pVI2373), assayed on 0.3% soft agar LB motility plates containing the indicated amount of L-arabinose as inducer. Graphed values are averages with standard deviations calculated from three independent colonies and were normalized by setting the values of the double mutant harbouring the vector control as 1. *P*-values, calculated with two-tailed student *t*-test (***P < 0.001), are relative to the vector control. Images are of corresponding protein expression levels as revealed by separation of 10 µg of soluble protein and subsequent Western analysis. Cropped images are derived from the same experiment processed in parallel on the same gel, and are shown alongside molecular size markers in Fig. S4B. (**B**) Relative swimming motility of *P. putida* KT2701-Δ*pp2258/*Δ*pp4397*, carrying *araC*/P_*BAD*_ expression plasmid for PP4397-FLAG (pVI2370) or YcgR-FLAG (pVI2373) as under panel A. Full images of the corresponding Western analysis for the cropped images are shown alongside molecular size markers in Fig. S4B.

To ascertain if PP4397 and YcgR showed cross-species functionality, plasmids expressing PP4397-FLAG and *E. coli* YcgR-FLAG under control of an *araC*/P_*BAD*_ promoter were introduced into *E. coli* MG1655-*ΔyhjH/ΔycgR* (which has elevated c-di-GMP levels due to the lack of the PDE YhjH*)* and *P. putida* Δ*pp2258/*Δ*pp4397* (which also has elevated c-di-GMP levels due to the lack of PP2258). Relative swimming motilities were assayed on LB soft 0.3% agar swimming motility plates containing 0 to 1.0% L-arabinose. As anticipated, motility of the *E. coli ΔyhjH/ΔycgR* strain was greatly reduced by expression of YcgR-FLAG induced with either 0.2% or 1% L-arabinose, but not by expression of PP4397-FLAG (Fig. 5A). However, Western analysis revealed that expression levels of PP4397-FLAG were notably lower than those of YcgR-FLAG, which likely underlies the inability of PP4397 to cause an altered motility phenotype in this strain (expanded Western Fig. S4B). In marked contrast, both YcgR-FLAG and PP4397-FLAG greatly reduced motility of *P. putida Δpp2258/Δpp4397* when expressed at similar levels (induced with 1% L-arabinose Figs 5B, and S4B). No reduction in motility was observed with c-di-GMP binding-deficient derivatives of either protein (PP4397-R127A-FLAG and its corresponding YcgR-R118A-FLAG counterpart, data not shown). These results lend strong support to the idea that despite their limited identity (Fig. 1), PP4397/FlgZ and YcgR are functional c-di-GMP responsive counterparts that act to control motility in *P. putida* and *E. coli*, respectively.

### PP4397-EYFP locates to the cytosolic compartment

*E. coli* are peritrichous, with flagella distributed throughout their surface, and fluorescently tagged YcgR has previously been found to localize to puncta on the cells together with the flagellar apparatus^17,19^. Similar puncta have been observed for *P. fluorescens* when fluorescently tagged FlgZ was overexpressed in cells with elevated c-di-GMP^23^. In the case of *P. aeruginosa*, which possesses a single polar flagellum, mono-copy fluorescently tagged FlgZ exhibits co-polar localization with the motility apparatus, and could be observed in a higher percentage of cells when c-di-GMP levels were elevated^24^. *P. putida* KT2701 used here possess a bundle of 6 to 10 flagella located at a single pole^27^ and, therefore, co-localization of its FlgZ/PP3497 counterpart with flagella would be anticipated to result in a polar localization.

To determine if PP4397, similarly to YcgR and other FlgZ counterparts, co-localizes with the flagellar machinery, PP4397-EYFP fusions were introduced into *P. putida*, both in mono-copy in its native location on the chromosome, and in multi-copy on an *araC*/P_*BAD*_ expression plasmid (as used in the motility assays in Fig. 5). Functionality of the PP4397-EYFP fusion, designed to have the same intervening residues as the YcgR-EYFP fusion, was confirmed by its maintenance of the reduced motility phenotype of the PP2258 null strain (Fig. S5). Western analysis was performed on cells harvested at the same time as cells were fixed for microscopy to facilitate correlation between images and corresponding protein expression levels.

**Figure 6:**
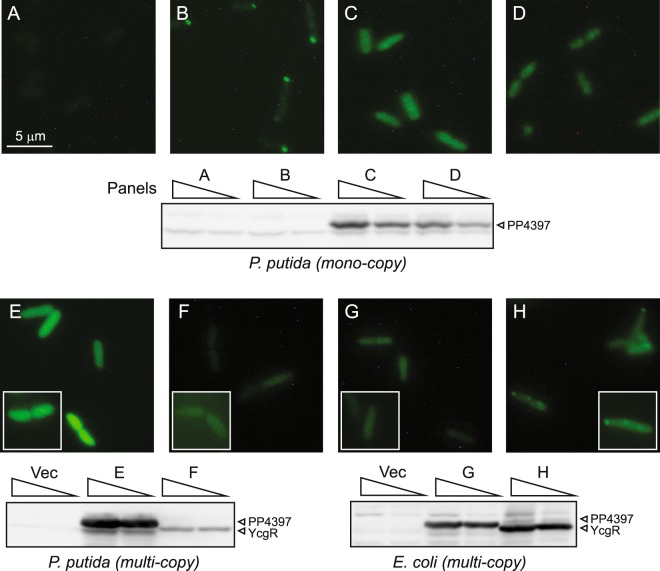
Localization of fluorescent proteins in *P. putida* and *E. coli* strains. Cells shown are representative of >6 fields viewed in two or three independent experiments. Upper panels (A to D) and cognate western analysis are of strains cultured on LB. (**A**) *P. putida* KT2701 (negative control; cells examined n = 735). (**B**) *P. putida* KT2701::*aer2-eyfp* (positive control, mono-copy chromosomal fusion; cells examined n = 342 of which 64% exhibited polar localization). (**C**) *P. putida* KT2701*::pp4397-eyfp* (mono-copy chromosomal fusion; cells examined n = 219). (**D**) *P. putida* KT2701::*pp4397-eyfp/Δpp2258* (PP2258 null with elevated c-di-GMP; cells examined n = 649). Western analysis of EYFP-tagged proteins expressed from mono-copy chromosomal translational fusions present in 50 and 25 µg of crude extract. Cell were harvested for Western analysis at the same time as fixing for imaging (after 2 to 2.5 hrs of growth; OD_600_ 0.5 to 0.7), which contrasts those shown for the motility assays in Fig. 5 (harvested after 5 hr of growth; OD_600_ ~3.5). Note that Aer2-EYFP, although clearly visible at the pole of the cell in panel B, is expressed at much lower levels than PP4397-EYFP and is not detected at the exposure shown. The cropped image is derived from the same experiment processed in parallel on the same gel, and are shown alongside molecular size markers in Fig. S6A. Lower panels [E to H] and cognate western are of strains cultured on LB in the presence of 1% L-arabinose. Boxed images are differentially exposed cells for comparison of the presence or lack of puncta. (**E**) *P. putida* KT2701-Δ*pp2258/*Δ*pp4397* (double PP2258/PP4397 null strain) carrying the multi-copy *araC*/P_*BAD*_ *pp4397-eyfp* expression plasmid (pVI2374). Cells examined: n = 271, 0% with puncta. (**F**) *P. putida* KT2701-Δ*pp2258/*Δ*PP4397* carrying the multi-copy *araC*/P_*BAD*_ *ycgR-eyfp* expression plasmid (pVI2375). Cells examined n = 282, 0% with puncta. (**G**) *E. coli* MG1655-*ΔyhjH/ΔycgR* (double YhjH/YcgR null strain) carrying the multi-copy *araC*/P_*BAD*_ *pp4397-eyfp* expression plasmid (pVI2374). Cells examined: n = 343, 0% with puncta. (**H**) *E. coli* MG1655-*ΔyhjH/ΔycgR* carrying the multi-copy *araC*/P_*BAD*_ *ycgR-eyfp* expression plasmid (pVI2375). Cells examined: n = 252, 25% with puncta [1 to 3 per cell]. Western analysis of EYFP-tagged proteins expressed from multi-copy translational fusions present in 25 and 12.5 µg of crude extract from *P. putida* (left) and *E. coli* (right). Cropped images are derived from the same experiment processed in parallel on the same gel, and are shown alongside molecular size markers in Fig. S6B.

In contrast to a mono-copy polar localization control (Aer2-EYFP^13^), mono-copy PP4397-EYFP was expressed at a higher level and localized to the cytoplasmic compartment in *P. putida* (Fig. 6, compare B to C). This apparent cytoplasmic localization was maintained in strains lacking PP2258 (Fig. 6, compare C and D) – i.e. under elevated c-di-GMP levels that results in altered swimming and swarming motility (Figs 3 and S5). This contrasts data for *P. fluorescens* and *P. aeruginosa*^23,24^, where cytosolic FlgZ counterparts could be visualized as puncta or at the pole under conditions where cellular c-di-GMP levels were elevated.

Cytosolic localization was also observed with PP4397-EYFP expressed from a multi-copy plasmid under inducing (1% L-arabinose) conditions (Fig. 6, compare C and E), which also alter *P. putida* motility (Figs 3 and S6C). While present at lower levels, multi-copy expression of YcgR-EYFP likewise showed a cytoplasmic location in *P. putida* (Fig. 6, compare E and F) and had a corresponding reduced effect on motility (Fig. S6C). This contrasts its punctate localization in *E. coli*, where it is expressed at similar levels as PP4397 (Fig. 6, compare F and H).

Taken together, the data in Figs 5 and 6 suggests that interaction between PP4397 (and likely YcgR) with the flagella motility apparatus of *P. putida* is weaker and/or more transient than that of YcgR with the motility apparatus of *E. coli*; and further, that a constant strict association with the flagella motor is not required for functionality. Although the interaction target of PP4397/FlgZ is unknown, based on the findings with the highly homologous FlgZ counterpart of *P. aeruginosa*^23^, it appears likely that one predominant target would be MotC and that functional replacement by YcgR relies on regions bearing common features between *P. putida* MotC and *E. coli* MotA proteins. Determining the interaction partner(s) for PP4397 is the subject of future studies.

## Concluding Remarks

As for other bacteria, artificial increase of c-di-GMP levels by expression of native or heterologous DGCs results in reduced flagella-mediated motility in *P. putida* KT2440^12^. Here we identify the PilZ domain containing PP4397/FlgZ protein as the effector relay protein that responds to elevated c-di-GMP levels resulting from lack of the signalling protein PP2258. Because *P. putida* harbours multiple c-di-GMP turnover proteins, it is likely that other c-di-GMP signalling pathways could also feed in to fine tune flagella performance through PP4397/FlgZ. Amongst the forty two *P. putida* c-di-GMP turnover proteins, PP2258 is the only one currently identified to possess both c-di-GMP degrading (PDE) and synthesising (DGC) activities^12^. However, the mechanism that controls the two opposing activities of PP2258 is unknown. One possibility is suggested by the genetic context of the *pp2258* gene, which is located in a bicistronic operon downstream of *aer1* that encodes a polar-localized receptor^13^. Both PP2258 and Aer1 possess PAS domains that are renowned for facilitating protein-protein interactions. Because the PAS domain of PP2258 is critical for its DGC activity^12^, it appears plausible that direct or indirect interaction between Aer1 and PP2258 could trigger a switch in its activities. Our current dissection of the signal transduction cascade from PP2258 to PP4397/FlgZ should greatly facilitate future work to determine if Aer1 controls PP2258 c-di-GMP signalling to ultimately control the ability of PP4397/FlgZ to act as an active hand-brake on the flagella motor.

## Methods

### Bacterial strains, growth conditions and general procedure

*E. coli* and *P. putida* strains (Table S1) were grown at 37 °C and 30 °C, respectively. *E. coli* DH5^30^ was used for construction and maintenance of expression plasmids. The specialised replication-permissive *E. coli* S17λ*pir* host, which expresses the Pir protein essential for replication of R6K^31^ was used for maintenance and conjugation of R6K-based suicide plasmids. *P. putida* strains used are all based on the genome sequenced KT2440^32^ or a spontaneous streptomycin resistant derivative of KT2440 (KT2701^33^). Plasmids (Table S2) were constructed by standard molecular techniques, as detailed in supporting information, and were introduced into *P. putida* by either electroporation or conjugation. Strains were cultured in Luria-Bertani (LB) broth (AppliChem GmbH) or on agar solidified plates supplemented with appropriate antibiotics. Concentrations used for *E. coli* were carbenicillin (Cb) 100 µg/ml, kanamycin (Km) 50 µg/ml, and tetracycline (Tc) 5 µg/ml, while those for *P. putida* were Cb 1 mg/ml, Km 50 µg/ml, and Tc 50 µg/ml.

### PCR determination of the genome organisation of pp4397

Generation of cDNA from total RNA isolated from *P. putida* was as previously described^13^. After cDNA synthesis, mRNA was removed by 15 min incubation at 37 °C in the presence of 0.23 M NaOH and then neutralized by adding HEPES to a final concentration of 625 mM. The cDNA was subsequently buffer exchanged to 10 mM Tris-HCl (pH 8.5) using High Pure PCR product purification kit (Roche) before being subjected to PCR using the primer sets listed in Table S3 and depicted in Fig. 2B.

### Generation of P. putida strains lacking PP4397

The *pp4397* gene replacement cassette (Δ*pp4397*::Tc) was introduced into the chromosome of *P. putida* KT2701 and its PP2258 null derivative^13^ via conjugation of pVI2299 (Table S2) from *E. coli* S17λ*pir* and subsequent double-site recombination as previously described^13^. Growth in medium containing Tc and 10% sucrose was used to select for recombinants. Diagnostic PCR of the resulting strains was used to confirm loss of the native intact *pp4397* gene and the presence of a fragment encompassing novel junctions of the Tc gene replacement and DNA upstream and downstream of the gene fragment of the suicide plasmid.

### Generation of P. putida mono-copy chromosomal transcriptional and translational fusions

Fusions were introduced into the chromosome of *P. putida* strains via single site recombination as previously described^13^. Suicide plasmids carrying 3′-regions of target genes with cognate transcriptional fusions to either the promoter-less *luxAB* genes or in-frame translational fusions to *eyfp*, were introduced by conjugation as described above. Recombinants were selected using the antibiotic resistance marker(s) of the vector. Since the suicide plasmids carry only 3′-portions of the target genes, the resulting strains contain one functional (fused) copy and one inactive truncated copy of the gene separated by plasmid DNA. Diagnostic PCR was used to confirm correct recombination using primers homologous to DNA upstream of the gene fragment on the suicide plasmid and the DNA of the fusion partner.

### *In vivo* luciferase transcriptional reporter assay

*P. putida* strains harboring mono-copy transcriptional fusions to *luxAB* were cultured in LB supplemented with appropriate antibiotics. To ensure balanced growth, overnight cultures were diluted in pre-warmed media and cultured into the exponential phase prior to a second dilution (to OD_600_ ~ 0.04) and initiation of the experiment. Growth and luciferase activity were monitored every 45 minutes for >9 hrs. Light emission was determined using 100 μl of culture after addition of decanal (1:2000 dilution) using a Infinite M200 (TECAN) luminometer.

### *In vitro* transcription assays

Single-round transcription assays were performed at 30 °C using *P. putida* KT2440-derived core RNA polymerase (10 nM) and σ^FliA^ (40 nM) as previously described^27^ with 10 nM supercoiled pTE103-based plasmids as DNA templates (Table S2). Assays (20 μl) were performed in T-buffer (35 mM Tris-Ac pH 7.9, 70 mM KAc, 5 mM MgAc_2_, 20 mM NH_4_Ac, 1 mM DTT and 0.275 mg/ml BSA). For holoenzyme formation, core RNA polymerase and σ^FliA^ were pre-incubated for 5 minutes prior to addition of template DNA and a further 20 minutes incubation to allow open-complex formation. Transcription was initiated by the addition of NTPs (final concentration: ATP, 500 μM; GTP and CTP, 200 μM each; UTP, 80 μM and [α-^32^P]-UTP (5 μCi at >3,000 Ci/mmol) in the presence of heparin (0.1 mg/ml) to prevent re-initiation. After a further 10 minutes at 30 °C, reactions were terminated by adding 5 μl of a stop/load mix (150 mM EDTA, 1 M NaCl, 14 M urea, 3% glycerol, 0.075% (w/v) xylene cyanol, 0.075% (w/v) bromophenol blue) and transcripts analysed on 7 M urea/5% (w/v) polyacrylamide sequencing gels. Radioactivity was quantified using a Storm 860 imaging system (Molecular Dynamics).

### Motility swimming and swarming plate assays

*E. coli* and *P. putida* strains were inoculated in LB supplemented with appropriate antibiotics and grown overnight. The next day, cultures were grown into early exponential phase, diluted to an OD_600nm_ of 0.1 and grown once again for 5 hours. Cultures were then adjusted to an OD_600nm_ of 0.3 and 5 μl were spotted on 0.3% soft agar LB plates for swimming assays and 0.5% agar LB plates for swarming assays. The resulting ring sizes were recorded after 6 h (*E. coli*) or 15 h (*P. putida*) of growth. Cells for western analysis were harvested at the same time as the dilutions prior to plating i.e. (after 5 hr of growth, OD_600_ = ~3.5).

### Western analysis

Cell pellets were washed and resupended in ice-cold sonication buffer (20 mM Tris-HCl pH 7.5, 0.2 mM NaCl, 1 mM EDTA) containing protease inhibitors (Complete EDTA-free protease inhibitor tablet; Roche). Cells were disrupted by sonication and samples subsequently clarified by centrifugation. Protein concentrations of the resulting crude extracts were determined with PIERCE BCA protein assay (Thermo Scientific). Soluble protein samples were separated by 12% SDS-PAGE and transferred to PVDF membranes (Amersham Hybond-P) by electro-transfer. FLAG-tagged and EYFP-tagged proteins were detected using monoclonal mouse M2 anti-FLAG (Kodak) and anti-GFP (Invitrogen) antibodies, respectively. Antibody-decorated bands were revealed using polyclonal secondary goat anti-mouse antibodies conjugated with HRP and ECL Plus Western Blotting Reagents (GE Healthcare). Results were recorded using AGFA Curix Ultra UV-G medical X-ray film (Figs 4 and S4A) or LAS 4000 imaging system (Fujifilm; Figs 5, 6, S4B and S6).

### Fluorescence microscopy

*E. coli* and *P. putida* strains were grown to exponential phase in LB, pelleted, washed and then fixed using paraformaldehyde (final concentration of 3%). Cells were adjusted to OD_600nm_ of 0.8 in 50% PBS (68.5 mM NaCl, 1.35 mM KCl, 5 mM Na_2_HPO_4_, 0.9 mM KH_2_PO_4_; Applichem) containing 1 mg/ml BSA. Culture aliquots (3 µl) were air dried on glass slides prior to coating with Mowiol 4–88 (Calbiochem) mounting media (10% Mowiol 4–88 [w/v], 25% glycerol, 0.1 M Tris-HCl pH 8.5). Cells were imaged using an Eclipse 90i (Nikon) microscope equipped with a Hamamatsu ORCA-ER CCD camera, using oil immersion and a 100x objective with a numerical aperture of 1.30. Cells for western analysis were harvested at the same time as for fixing, i.e. mid exponential phase (OD_600_ = ~0.5 to 0.7), after 2 to 2.5 hours of growth.

### Data availability statement

All data generated or analyzed during this study are included in this article and its Supplementary Information file.

## Electronic supplementary material

Supplementary Information
