# Supplementary material for: PP4397/FlgZ provides the link between PP2258 c-di-GMP signalling and altered motility in Pseudomonas putida
Source: Sci Rep. 2018 Aug 15;8:12205. doi: 10.1038/s41598-018-29785-w (PMC6093933; doi:10.1038/s41598-018-29785-w)
Supplement: Supplementary file 1 — Supplementary Information [file 41598_2018_29785_MOESM1_ESM.pdf]

Supplementary Information

PP4397/FlgZ provides the link between PP2258 c-di-GMP signalling and altered motility in *Pseudomonas putida*

Lisa Wirebrand[1], Sofia Österberg[1], Aroa López-Sánchez[2], Fernando Govantes[2] and Victoria Shingler[1*]

[1]*Department of Molecular Biology, Umeå University, SE-901 87 Umeå, Sweden*
[2]*Departamento de Biología Molecular e Ingeniería Bioquímica, Universidad Pablo de Olavide, Sevilla, Spain.*

Supplementary Methods

Plasmid constructions: Plasmids (Table S2) were constructed by standard molecular techniques with the fidelity of all PCR amplified DNA confirmed.

P_{flgM} *in vitro* transcription plasmid: Plasmid pVI2368 for *in vitro* transcriptional assay from P_{flgM} was constructed by introducing a custom synthesized EcoRI to BamHI fragment (GenScript) encompassing -185 to +2 relative to the A of the ATG initiation codon of *flgM*, between the EcoRI and BamHI sites of pTE103.

Δ*pp4397::*Tc gene replacement/insertion suicide plasmid: Plasmid pVI2299, carrying a tetracycline resistance *pp4397* gene replacement cassette on the pDM4-N suicide plasmid, was constructed by a step-wise procedure. First, a 240 bp NotI-SacI fragment (encompassing upstream DNA and the first 12 codons of *pp4397*) and a 192 bp SacI-XhoI fragment (encompassing the last 35 codons of *pp4397* and downstream DNA) were PCR amplified (Table S4). These fragments were then assembled between the NotI and XhoI sites on a derivative of pBluescript SK (Stratagene) manipulated to lack the SacI site of its polycloning region (pVI2199). Next, a SacI fragment carrying the Tc resistance gene from p34S-Tc was introduced into the unique SacI restriction site, orientated so transcription of the Tc gene was in the same direction as that of *pp4397*. Finally, the NotI-XhoI gene replacement cassette (with the Tc gene replacing the internal 200 codons of *pp4397*) was cloned between the same sites of the pDM4-N suicide vector.

Translational fusion of *pp4397* to *eyfp*: The 3´-end of *pp4397* was amplified as a 356 bp XhoI/HindIII fragment (Table S4) and inserted between the XhoI and HindIII sites of pBS-eyfp[13],

upstream of the e*yfp* gene. The resulting 1156 bp XhoI/BamHI 3´-*pp4397-eyfp* translational fusion was then cloned between the XhoI and BglII sites of suicide plasmids pDM4-Km3 and pDM4-Tc to give pVI2377 and pVI2378, respectively. These plasmids were used to create mono-copy *pp4397-eyfp* fusions on the *P. putida* chromosome.

Transcriptional fusion of *pp4397* to *luxAB*: Plasmid pVI2376, carrying a 3´-*pp4397-luxAB* transcriptional fusion on suicide vector pTc805L, was generated using the same XhoI/HindIII 3´-*pp4397* region as above but rendered blunt ended at the HindIII site, and cloning between the XhoI/StuI sites of pTc805L, upstream the promoter-less *luxAB* genes of the vector. This plasmid was used to create mono-copy *pp4397-luxAB* transcriptional fusions on the *P. putida* chromosome.

***lacI*^{Q}/P$_{tac}$ expression plasmids:** Plasmid pVI2301 carrying *pp4397* under the control of the *lacI*^{Q}/P$_{tac}$ expression system was constructed by PCR amplification of the *pp4397* coding region as a NotI to XhoI fragment (Table S4) and cloned between these sites of the expression vector pVI2300. R127A and G162A derivatives carried on plasmids pVI2302 and pVI2303, respectively, were generated in a similar manner but with the cognate substitutions introduced by overlapping PCR (Table S4). Plasmids expressing FLAG-tagged versions of PP4397 and its derivatives under control of *lacI*^{Q}/P$_{tac}$ were constructed in a two-step process. First, the 5´-end of wild type and mutant versions of *pp4397* (as HindIII-NcoI fragments) and the 3´-end of *pp4397* fused to the eight codons for the FLAG-tag (as a PCR generated NcoI-EcoRI fragment, Table S4) were assembled on pBluescript SK (Stratagene). The HindIII-EcoRI fragments encompassing the *pp4397-FLAG* fusions were then cloned downstream of the *lacI*^{Q}/P$_{tac}$ expression system of pVI2300, generating pVI2304 to pVI2306.

***araC*/P$_{BAD}$ expression plasmids:** Plasmids pVI2370 to pVI2372, carrying *pp4397-FLAG* derivatives under control of an *araC*/P$_{BAD}$ expression system, were constructed by PCR amplification (Table S4) of the coding region as a NdeI-HindIII fragments using pVI2304 to pVI2306 as templates, with subsequent cloning between these sites of vector pVI2198. Cognate expression plasmids for *E. coli ycgR-FLAG,* (pVI2373) and *ycgR-R118A-FLAG* (pVI2379), were generated using custom synthesised NdeI-HindIII fragments (GenScript) cloned between these sites of pVI2198.

Plasmids for multi-copy expression of PP4397-EYFP (pVI2374) and YcgR-EYFP (pVI2375) translational fusions were constructed step-wise. For *pp4397-eyfp,* the *pp4397* gene was first reconstructed by cloning the 5´-end of *pp4397* (as a NdeI-NcoI fragment from pVI2370) and the remaining 3´-end of *pp4397* as a NcoI-NheI fragment (custom synthesized GenScript) on pVI2199. An in-frame fusion to *eyfp* (identical to that of pVI2377 and pVI2378) was subsequently generated by cloning the NheI-BamHI e*yfp*-gene fragment from pBS-eyfp at the 3´-end of *pp4397*. Finally, the

NdeI-BamHI fragment spanning *pp4397-eyfp* was introduced downstream of the *araC*/P_{BAD} expression system of pVI2198, resulting in pVI2374. For *ycgR-eyfp*, the same NheI-BamHI e*yfp*-gene fragment was first introduced into pVI2199 prior to in-frame reconstruction of *ycgR* upstream to create an analogous 3´-translational fusion. The *ycgR* gene was reconstructed by sequential cloning of the 5´-region (as a NdeI-XhoI fragment from pVI2373) and a double stranded XhoI-NheI linker (Table S4) encompassing the 3´-end of *ycgR* to create an identical fusion to *eyfp* as in pVI2374. Similarly, the resulting *ycgR-eyfp* fusion was then introduced as a NdeI-HindIII fragment between these sites of vector pVI2198, generating pVI2375.

Biofilm growth and dispersal: Biofilm growth and dispersal assays were performed using serial dilutions of overnight cultures grown in LB as previously described[29]. Overnight cultures were first adjusted to an OD_{600} of 0.1 and then 150 µl of a serial dilution of these cultures in LB were dispensed into wells of Costar 96 microtiter polystyrene plates (Corning). Plates were incubated at 25°C with moderate shaking (150 rpm) for the indicated period of time, after which they were processed for planktonic (OD_{600}) and biofilm growth (crystal violet staining of well-associated cells) essentially as described[34]. Note that although plates are incubated for a fixed period of time, the 10-fold dilutions result in cultures at different stages of growth to recapitulate a time course for biofilm formation and dispersal. For each experiment, three biological replicates were assayed in sextuplicate.

Table S1. Bacterial strains.

E. coli	Properties	Reference
DH5	K12 general cloning strain; *endA1, hsdR17*(r_K^- m_K^+), *supE44, thi-1, recA1, gyrA96, relA1,* ϕ80d *lacZ* ΔM15	(30)
S17-1λ*pir*	Sm^R, Tp^R; specialized auxotrophic strain expressing the Pir protein (required for replication of R6K-based suicide plasmids) and mobilization functions on the chromosome.	(31)
MG1655-Δ*yhjH/*Δ*ycgR*	K12 derivative, YhjH/YcgR double null (elevated c-di-GMP)	(17)

P. putida	Properties	Reference
KT2440	Genome sequenced *P. putida* wild-type strain	(32)
KT2440-*fliA*::Km	Km^R; FliA (σ^{FliA}) null insertion derivative of KT2440	(37)
KT2440-*rpoN*::Km	Km^R; RpoN (σ^{54}) null insertion derivative of KT2440	(38)
KT2701	Spontaneous Sm^R derivative of KT2440	(33)
KT2701-Δ*aer1*	Sm^R; Aer1 null derivative of KT2701	This study
PP2167	Sm^R, Km^R, Aer2-EYFP (mono-copy) expressed from its native location in KT2701	(13)
PP3467	Sm^R, Km^R, Δ*pp2258::*Km; PP2258 null derivative of KT2701 (elevated c-di-GMP)	(13)
PP3468	Sm^R, Km^R, Tc^R, Δ*pp2258*::Km/Δ*pp4397::*Tc; double PP2258 and PP4397 null derivative of KT2701	This study
PP3469	Sm^R, Tc^R, Δ*pp4397::*Tc; PP4397 null derivative of KT2701	This study
PP3617	Sm^R, Km^R, PP4397-EYFP (mono-copy) expressed from its native location in KT2701	This study
PP3669	Sm^R, Km^R, Tc^R, PP4397-EYFP expressed from its native location in a KT2701 PP2258 null background (elevated c-di-GMP)	This study
PP3733	Tc^R, P_{flgM}-*luxAB* mono-copy transcriptional reporter in KT2440; 3´-fusion to *pp4397*	This study
PP3734	Tc^R, Km^R, P_{flgM}-*luxAB* mono-copy transcriptional reporter in KT2440-FliA (σ^{FliA}) null; 3´-fusion to *pp4397*	This study
PP3735	Tc^R, Km^R, P_{flgM}-*luxAB* mono-copy transcriptional reporter in KT2440-RpoN (σ^{54}) null; 3´-fusion to *pp4397*	This study

Abbreviations: Cm, chlorampheincol; Km; kanamycin; Tc, tetracycline; Sm, streptomycin; Tp, trimethoprim.

Table S2. Plasmids used in this study.

**Vectors**	**Properties**	**Reference/source**
pBluescript SK	Cb^R, Standard cloning vector	Stratagene
pVI2199	Cb^R, derivative of pBluescript SK with an altered polyclonal site: SacI/SacII/NotI/XbaI/NdeI/BglII/ClaI/XhoI/ NcoI/EcoRI/XmaI/SmaI/NheI/StuI/SpeI/BamHI/HindIII/KpnI	This study
pBS-eyfp	Cb^R, *eyfp* gene as a HindIII to BamHI fragment on pBluescript SK	(13)
p34S-Tc	Source of the Tc^R gene used in strain constructions	(39)
pDM4	Cm^R, R6K-based suicide plasmid carrying *sacB* for sucrose selection of double site recombinants	(40)
pDM4-Km3	Cm^R, Km^R, pDM4 R6K-based suicide plasmid	(13)
pDM4-N	Cm^R, pDM4 derivative with the polylinker SalI site replaced by a unique NotI site	This study
pDM4-Tc	Cm^R, Tc^R, pDM4 R6K-based suicide plasmid	(13)
pTc805L	Tc^R, R6K-based suicide plasmid carrying promoterless *luxAB* genes	(13)
pMMB66HE	Cb^R, IncQ broad host range *lacI*^{Q}/P_{tac} expression vector	(41)
pVI533	Cb^R, IncQ broad host range *araC/*P_{BAD} expression vector	(42)
pVI2198	Cb^R, derivative of pVI533 with an altered polyclonal site downstream of the *araC/*P_{BAD} expression system: NdeI/SmaI /SalI/PstI/HindIII	This study
pVI2300	Cb^R, derivative of pMMB66HE with an altered polyclonal site downstream of the *lacI*^{Q}/P_{tac} expression system: HindIII/NotI/SacI/XhoI/PstI/SmaI/KpnI/XbaI/BglII/EcoRI	This study
pTE103	Cb^R, *in vitro* transcription vector; carries a strong T7 transcriptional terminator downstream of the polycloning site.	(43)
***In vitro* transcription plasmids**		
pVI1011	Cb^R, carrying P_{aer2} on pTE103; -180 to +2 relative to the A of the ATG initiation codon of *aer2*	(27)
pVI2368	Cb^R, carrying P_{flgM} on pTE103; -185 to +2 relative to the A of the ATG initiation codon of *flgM*	This study
**Gene replacement R6K-based plasmid**		
pVI2299	Cm^R Tc^R, Δ*pp4397*::Tc as a NotI-XhoI cassette on pDM4-N	This study

**Transcriptional and translational fusions on R6K-based suicide plasmids**		
pVI2376	Tc^R, 3´-*pp4397-luxAB* on pTc805L	This study
pVI2377	Cm^R, 3´-*pp4397-eyfp* on pDM4-Km3	This study
pVI2378	Cm^R, 3´-*pp4397-eyfp* on pDM4-Tc	This study
**Expression plasmids**		
pVI814	Cb^R, $lacI^Q$/P_{tac}-*aer1*	(13)
pVI2301	Cb^R, $lacI^Q$/P_{tac}-*pp4397* on pVI2300	This study
pVI2302	Cb^R, $lacI^Q$/P_{tac}-*pp4397*-R127A on pVI2300	This study
pVI2303	Cb^R, $lacI^Q$/P_{tac}-*pp4397*-G162A on pVI2300	This study
pVI2304	Cb^R, $lacI^Q$/P_{tac}-*pp4397*-FLAG on pVI2300	This study
pVI2305	Cb^R, $lacI^Q$/P_{tac}-*pp4397*-R127A-FLAG on pVI2300	This study
pVI2306	Cb^R, $lacI^Q$/P_{tac}-*pp4397*-G162A-FLAG on pVI2300	This study
pVI2370	Cb^R, *araC/*P_{BAD}-*pp4397*-FLAG on pVI2198	This study
pVI2371	Cb^R, *araC/*P_{BAD}-*pp4397*- R127A-FLAG on pVI2198	This study
pVI2372	Cb^R, *araC/*P_{BAD}-*pp4397*- G162A-FLAG on pVI2198	This study
pVI2373	Cb^R, *araC/*P_{BAD}-*ycgR*-FLAG on pVI2198	This study
pVI2379	Cb^R, *araC/*P_{BAD}-*ycgR*-R118A-FLAG on pVI2198	This study
pVI2374	Cb^R, *araC/*P_{BAD}-*pp4397-eyfp* on pVI2198	This study
pVI2375	Cb^R, *araC/*P_{BAD}-*ycgR-eyfp* on pVI2198	This study

Abbreviations: Cb, carbenicillin; Cm, chloramphenicol; Km, kanamycin; Tc, tetracycline.

Table S3. Primers used to determine the genome organisation of *pp4397*.

Primer pair[a]	Description	Sequences
(1) 2959/2960	Spanning *cheV-3* - *flgA*	f 5′-GCGGAACAGCAGCAATTCCAGGC-3′<br>r 5′-CGGGTTGACCTGGATTTCATAGC-3′
(2) 2907/2908	Spanning *flgA* - *flgM*	f 5′-GCAACCTCAATTCCAAACGC-3′<br>r 5′-GCACGCCGCCCGTGACGGAC-3′
(3) 3148/3149	Spanning *flgM* - *pp4396*	f 5′-CGCCAGCAAACTGCTTGATTT-3′<br>r 5′-CCGCTGCAGCATCACATCAC-3′
(4) 2911/2912	Spanning *pp4396* - *pp4397*	f 5′-GCCAGCAGGTCAACCAGACC-3′<br>r 5′-CGGTCGTGGAAGGTGATGATC-3′

[a] Numbers in brackets are the primer pair numbers as used in Fig. 2B. The f and r designations indicate forward and revers primer, respectively.

Table S4. Oligonucleotides and linkers used in plasmid constructions

PCR 2722/2723; amplification of the 240 bp NotI-SacI fragment of *pp4397* (pVI2299)

```
5′-CCGGGGCGGCCGCACGTCACCAACAACCAGATC-3′  2722 f (NotI underlined)
5′-CCGCGAGCTCGGTGGTTGCGGGGCATCGGATTC-3′  2723 r (SacI underlined)
```

PCR 2724/2725; amplification of the 192 bp SacI-XhoI fragment of *pp4397* (pVI2299)

```
5′-CGGCACCACCGAGCTCGGCGTGCGTTTCCACAAC-3′ 2724 f (SacI underlined)
5′-CGCGGCTCGAGCCGCGAAGCGGCCAGCCCAGC-3′   2725 r (XhoI underlined)
```

PCR 2889/2890; amplification of the 356 bp XhoI/HindIII fragment of *pp4397* (pVI2377 & pVI2378)

```
5′-CGCGCTCGAGGTCGACATCATCCTT-3′          2889 f (XhoI underlined)
5′-CCGCAAGCTTATAATCGTCCTTGTC-3′          2890 r (HindIII underlined)
```

PCR 2722/2725; amplification of NotI-XhoI regions of *pp4397* derivatives (pVI2301-pVI2303)

```
5′-CCGGGGCGGCCGCACGTCACCAACAACCAGATC-3′  2722 f (NotI underlined)
5′-CGCGGCTCGAGCCGCGAAGCGGCCAGCCCAGC-3′   2725 r (XhoI underlined)

used in conjunction with mutagenic primers in overlapping PCR:
for R127A
5′-CGCCTTCGCCGCCGCGCTGAAGCTATCGCAGC-3′    2886 f
5′-GCGCGGCGGCGAAGGCGTTGCGGCGCTGGTGGT-3′   2885 r
for G162A
5′-CGGCCACCGCCTGCAAACTGCGCTTCGAAGGC-3′    2888 f
5′-GCAGGCGGTGGCCGAGATATCCAGCAGCTTGCC-3′   2887 r
```

PCR 2968/2969; amplification of NcoI-EcoRI Flag-tagged region of *pp4397* (pVI2304-pVI2306)

```
5′-CTGGAAGCTTCCATGGTCGAGCTGCGTCATC-3′     2968 f (HindIII & NcoI underlined)
5′-GTGGCGAATTCTCACTTATCGTCATCGTCTTTATAATCGTCCTTGTCGAACCGC 2969 r (EcoRI underlined)
```

PCR 3009/3010; of amplification NdeI/HindIII regions of *pp4397* derivatives (pVI2370-pVI2372)

```
5′-CCAAGCATATGTTCAATGAATCCGATGC-3′        3009 f (NdeI underlined)
5′-CCCGAAGCTTCTCACTTATCGTCATCGTCT-3′      3010 r (HindIII underlined)
```

Linker 75 SalI compatible ends (site destroyed) in pDM4-N

```
5′-TCGAAGCGGCCGCT-3′      (introduced NotI underlined)
    3′-TCGCCGGCGAAGCT-5′
```

Linker 173 EcoRI compatible ends (site destroyed) in pVI2198

```
5′-AATTACATATGT-3′       (introduced NdeI underlined)
    3′-TGTATACATTAA-5′
```

Linker 3244/3245 SpeI and XhoI compatible ends (sites destroyed) in pVI2199

```
5′-CTAGCATATGAGATCTATCGATCTCGAGCCATGGAATTCCCGGGCTAGCAGGCCTACTAGTGGATCCAAGCTT-3′
    3′-GTATACTCTAGATAGCTAGAGCTCGGTACCTTAAGGGCCCGATCGTCCGGATGATCACCTAGGTTCGAAAGCT-5′
```

Linker 2291/2292 EcoRI and HindIII compatible ends (sites regenerated) in pVI2300

```
5′-AATTCAGATCTAGAGGTACCCGGGCTGCAGCTCGAGCTCGCGGCCGCA-3′
    3′-GTCTAGATCTCCATGGGCCCGACGTCGAGCTCGAGCGCCGGCGTTCGA-5′
```

Linker 3246/3247 XhoI and NheI compatible ends (sites regenerated) in pVI2375

```
5′-TCGAGCGAGAAGCCCGGGAAAAAGCGGACAAAGTGCGCGACAAACTTCCG-3′
    3′-CGCTCTTCGGGCCCTTTTTCGCCTGTTTCACGCGCTGTTTGAAGGCGATC-5′
```

The f and r designations indicate forward and revers primer, respectively.

Supplementary Figures

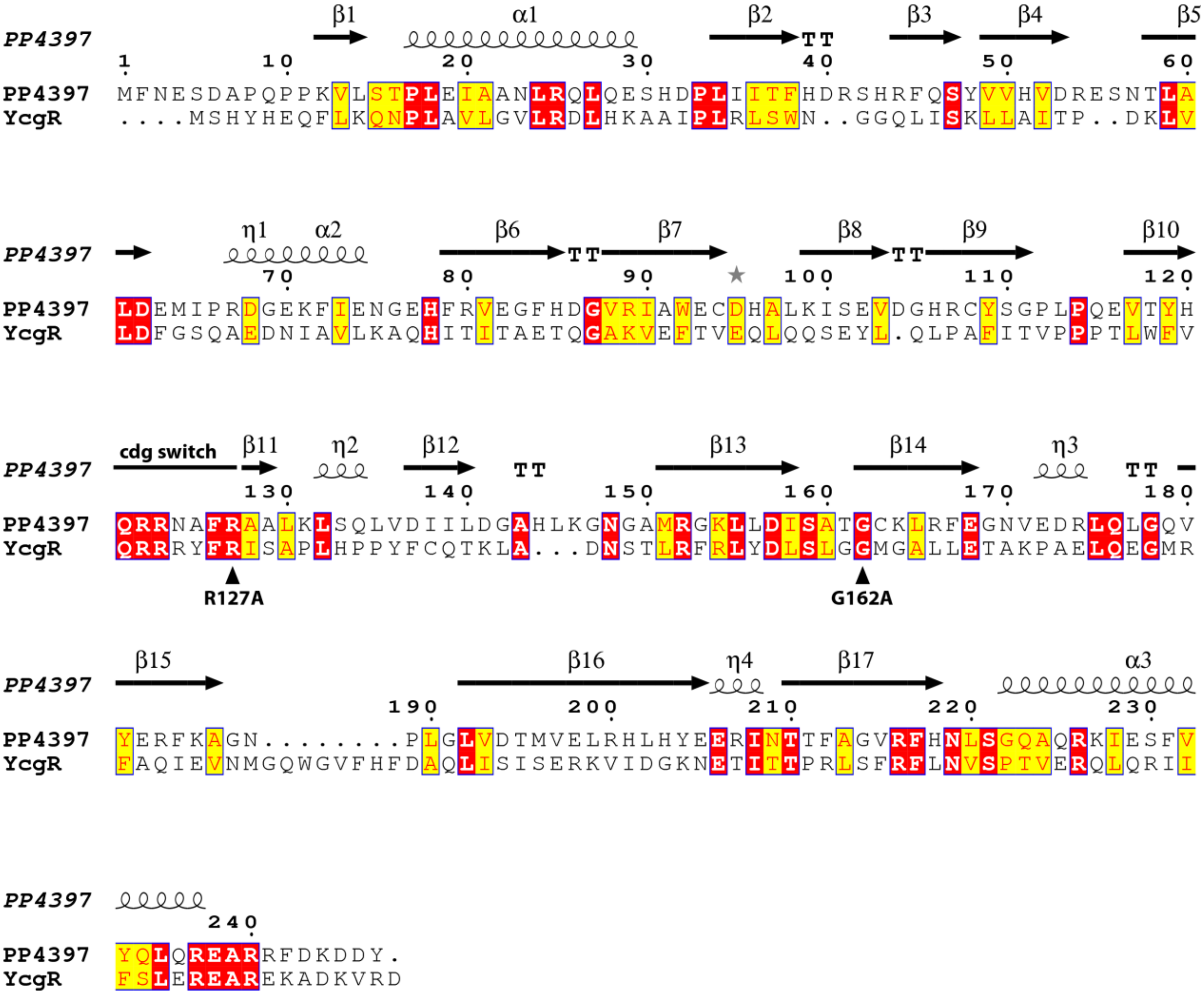

Fig. S1 Alignment of PP4397 and YcgR as in references 15 and 25 using ESPript[35]. Secondary structure of PP4397 is shown above. β-sheets are indicated as arrows, 3_{10}- (η) and α-helixes are shown as coils, while strict β-turns are indicated by T. The grey star marks a residue with alternative conformations. Red highlights indicate identical residues, while similar residues are highlighted with yellow. Residues mutated in R127A and G162A mutants are marked with triangles, while the c-di-GMP (cgd) switch region encompassing R127A is shown over bordered.

```
PP      -----------------MFNESDAPQPPKVLSTPLEIAANLRQLQESHDPLIITFHDRSH  43
PF      --------------MFNASNADDAPQPPKVLTTPLEIAGNLRMLQESHDPLIITFHERSQ  46
PA      MLSLRHSCPRRALKVPNPFVEEAGPQPPKVLKAPVEIQANLRLLQDSRDSLLITFADRNQ  60
                             . .*******.:*:** .*** **:*:* *:*** :*.:

PP      RFQSYVVHVDRESNTLALDEMIPRDGEKFIENGEHFRVEGFHDGVRIAWECDHALKISEV 103
PF      RFQSYLVNVDRETNSIALDEMIPRDGERFLLAGEPFRVEGFHDGVRIAWDGKGPLTIDES 106
PA      RFQSFLVDIDRDRGMVALDELIPNTGERFLQNGEAFRVEAFHEGVRIAWECERPVQFGEH 120
        ****::*.:**:   :****:**. **:*:  ** ****.**:******: .  : : *

PP      DGHRCYSGPLPQEVTYHQRRNAFRAALKLSQLVDIILDGAHLKGNGAMRGKLLDISATGC 163
PF      SDGRCYRGALPDEVVYHQRRNAFRAALKLAQLVSVELGGDKMK--SPVDGKLLDISATGC 164
PA      DGVPCYWAALPAEVLYHQRRNAYRASIKQSQPIAAEIAGEKLR--APLSGQLLDISATGA 178
        .   ** . ** ** *******:**::* :* :   : * :::  . : *:********.

PP      KLRFEGNVEDRLQLGQVYERFKAGNPLGLVDTMVELRHLHYEERINTTFAGVRFHNLSGQ 223
PF      KLRFDGDITERLQLGQVYERFIAALPFGNMTAPVELRYLHFEERINTTFAGVRFHNISGL 224
PA      KLRFAGNVAERLHPGEIYEDFSAQLPQGAISSAIELRHVRFDEKLDATFAGVRFSEMSGL 238
        **** *:: :**: *::** * *  * * : : :***:::::*::::******* ::**

PP      AQRKIESFVYQLQREARRFDKDDY  247
PF      VQRQVERFVYQLQREARRFDKDDL  248 [179/249(72%), 203/249(81%)]
PA      AQRQVDRFVYQLQREARRFEKDELF 263 [138/245(56%), 181/245(73%)]
        .**::: ************:**:
```

Fig. S2 Alignment of FlgZ proteins from three Pseudomonads

P. putida KT2440 (PP_4397; PP), *P. fluorescens* F113 (PSF113_4460; PF), *P. aeruginosa* PAO1and PA14 (PA3353 and PA14_02700; PA). Nine highly conserved residues of PilZ domains (bold and underlined) that encompass the RXXXR and (D/N)XSXXG motifs of type I c-di-GMP binding PilZ domains are shown for orientation. Figures in parenthesis give % identity and similarity to FlgZ/PP4397.

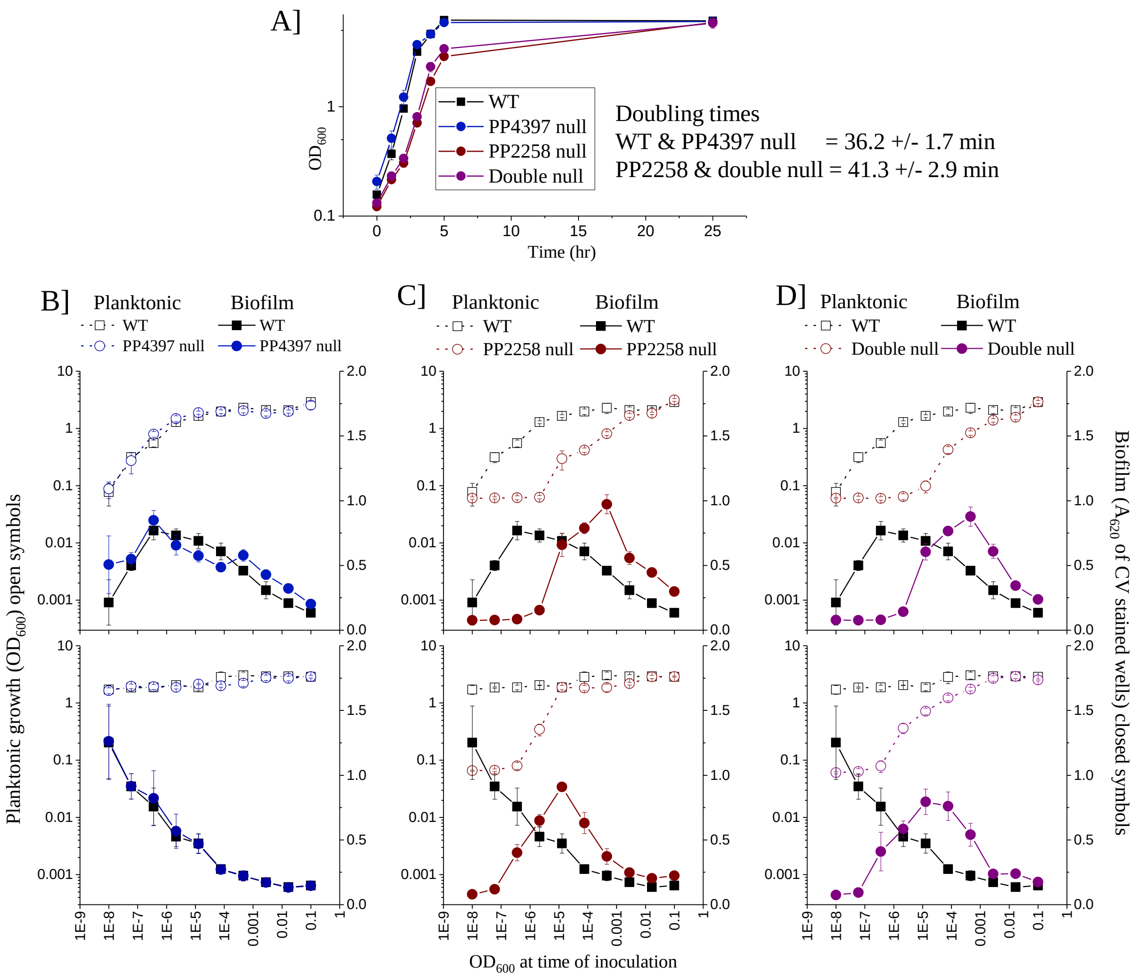

Fig. S3 **Growth curves and dilution series-based planktonic and biofilm assays of *P. putida* derivatives**

A] Growth curves of the indicated *P. putida* KT2701 derivatives on LB. Data are the average +/- standard deviation of six independent growth curves for each strain. Note that the cell were pre-grown to the exponential phase prior to dilution so do not show the longer lag phases of the PP2258 and PP2258/PP4397 double null strains.

B] to D] Planktonic (left axes, open symbols) or biofilm growth (right axes, closed symbols) is plotted against the initial A_{600} of each dilution. Black squares represent the wild-type strain while coloured circles are data from the indicated null strains. Prior to measurements, plates were incubated for 20 hours (upper panels) to assess biofilm formation, or 26 hours (lower panels) to visualize biofilm dispersal. Plots display one representative experiment of three biological replicates. Error bars are the standard deviation of the six technical replicates. Note that apart from a delay in the response, which presumably reflects different growth kinetics (longer lag and slower doubling times) of the PP2258 null and the PP2258/PP4397 double null strains, these strains accumulate and disperse biofilms in a similar manner to the wild-type (WT) and PP4397 / FlgZ null strains.

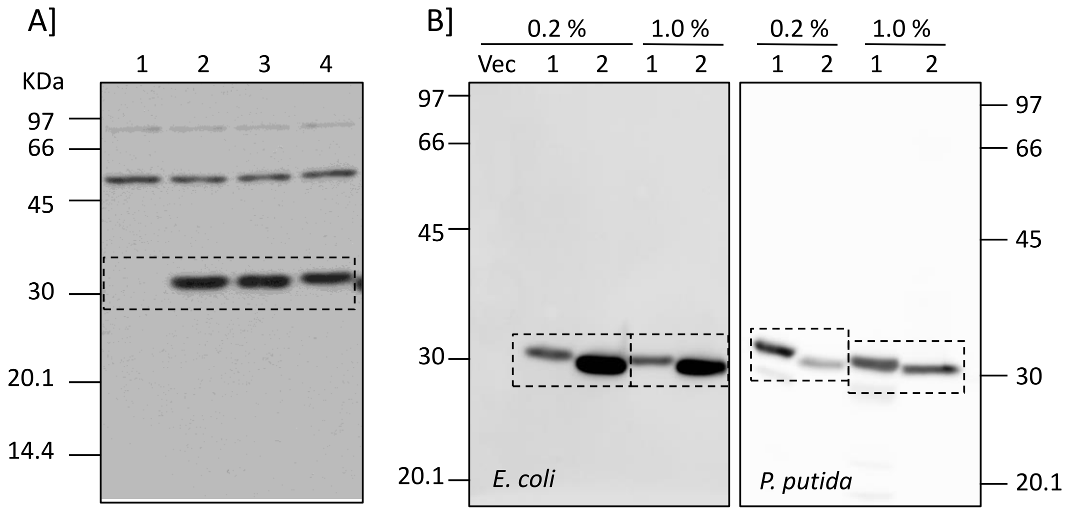

Fig. S4 **Levels of FLAG-tagged PP4397 and YcgR in strains used in Figs. 4 and 5**

A] Western analysis of the FLAG-tagged PP4397 derivatives present in 10 µg of crude extract as under Fig. 4; cropped regions shown in Fig. 4 are indicated by dashed boxes.

B] Western analysis of the FLAG-tagged PP4397 and YcgR present in 10 µg of crude extract of *E. coli* (left) or *P. putida* (right) carrying *araC*/P_{BAD} expression plasmid for *P. putida* PP4397-FLAG (1, pVI2370) or *E. coli* YcgR-FLAG (2, pVI2373), as under Fig. 5; cropped regions shown in Fig. 5 are indicated by dashed boxes.

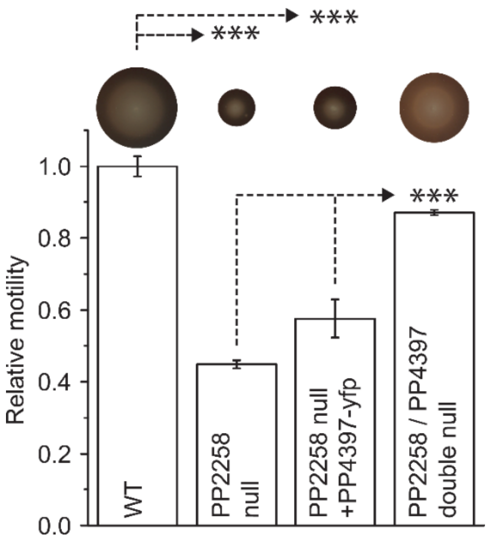

Fig. S5 **The motility defect of the *P. putida* PP2258 null is maintained by the PP4397-EYFP fusion protein**

Relative swimming motility of *P. putida* KT2701 derivatives assayed on 0.3% soft agar LB plates as under Fig. 3. Strains: wild type (WT), PP2258 null; PP2258 null with a monocopy chromosomal fusion of PP4397-EYFP; and the PP2258/PP4397 double null strain. Representative swim rings are shown above. Note that impairment of PP4397 function by the EYFP fusion would be anticipated to result in a phenotype similar to the PP2258/PP4397 double null strain. Data are the average +/- standard deviation of the three independent colonies, normalized by setting the swim ring size of the wild-type as 1. *P*-values shown for relevant comparisons were calculated with two-tailed student *t*-test (***$P < 0.001$).

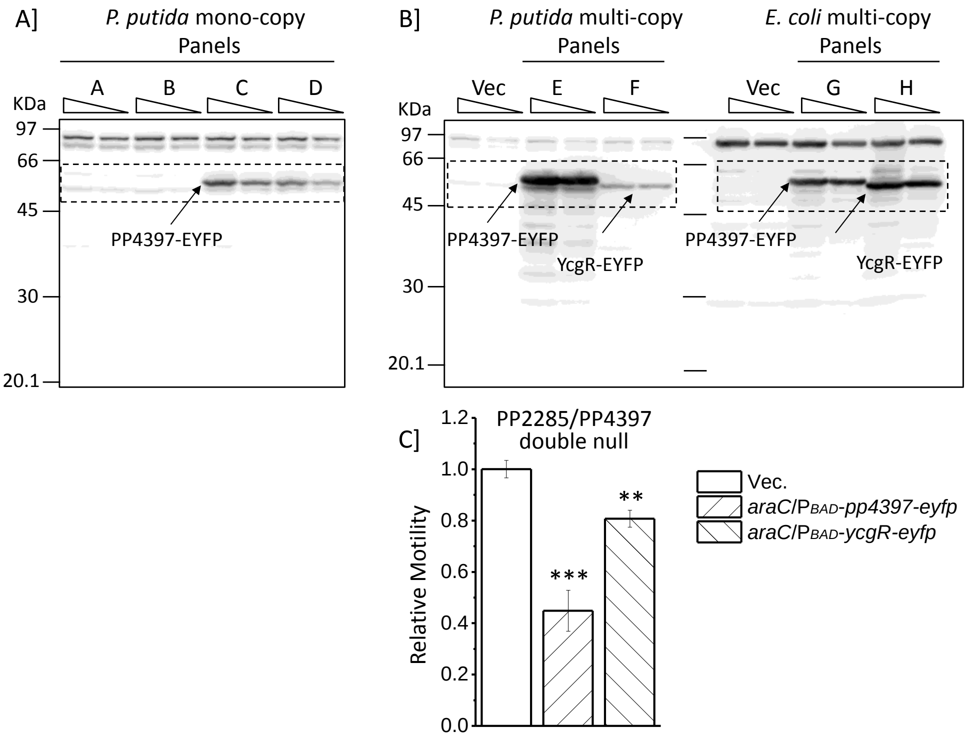

Fig. S6

A] Western analysis of the EYFP-tagged PP4397 expressed from mono-copy chromosomal translational fusions present in 50 and 25 µg of crude extract as under Fig. 6; cropped regions shown in Fig. 6 are indicated by dashed boxes.

B] As under panel A, but for EYFP-tagged proteins carried on multi-copy plasmids [16-20 copies per cell]. Expression from the plasmid *araC*/P_{BAD} was induced by the addition of 1% arabinose in the growth media. Western analysis is of 25 and 12.5 µg of crude extract from *P. putida* (left) and *E. coli* (right) as under Fig. 6; cropped regions shown in Fig. 6 are indicated by dashed boxes.

C] Relative swimming motility of the *P. putida* PP2258/PP4397 double null derivative, harbouring the indicated plasmids, assayed on 0.3% soft agar LB plates supplemented with carbenicillin and 1% arabinose. Data are the average +/- standard deviation of the three independent colonies, normalized by setting the swim ring size of the vector control strain as 1. *P*-values shown for comparisons to the vector control strain were calculated with two-tailed student *t*-test (****P* < 0.001, ***P* < 0.01).

Additional Supplementary References

36. O'Toole, G. A. *et al.* Genetic approaches to study of biofilms. *Methods Enzymol.* **310**, 91-109 (1999).

37. Rodriguez-Herva, J. J. *et al.* Physiological and transcriptomic characterization of a fliA mutant of *Pseudomonas putida* KT2440. *Environ. Microbiol. Rep.* **2**, 373-380 (2010).

38. Kohler, T., Harayama, S., Ramos, J. L. & Timmis, K.N. Involvement of *Pseudomonas putida* RpoN sigma factor in regulation of various metabolic functions. *J. Bacteriol.* **171**, 4326-4333 (1989).

39. Dennis, J. J. & Zylstra, G. J. Plasposons: modular self-cloning minitransposon derivatives for rapid genetic analysis of gram-negative bacterial genomes. *Appl. Environ. Microbiol.* **64**, 2710-2715 (1998).

40. Milton, D. L., O'Toole, R., Horstedt, P. & Wolf-Watz, H. Flagellin A is essential for the virulence of *Vibrio anguillarum*. *J. Bacteriol.* **178**, 1310-1319 (1996).

41. Furste, J. P. *et al.* Molecular cloning of the plasmid RP4 primase region in a multi-host-range tacP expression vector. *Gene* **48**, 119-131 (1986).

42. Sze, C. C. & Shingler, V. The alarmone (p)ppGpp mediates physiological-responsive control at the σ^{54}-dependent Po promoter. *Mol. Microbiol.* **31**, 1217-1228 (1999).

43. Elliott, T. & Geiduschek, E. P. Defining a bacteriophage T4 late promoter: absence of a "-35" region. *Cell* **36**, 211-219 (1984).
